# Factors Influencing the Degree of Disability in Patients With Multiple Sclerosis

**DOI:** 10.3389/fneur.2021.714631

**Published:** 2021-10-13

**Authors:** Huiru Xue, Zhenzhen Yang, Li Wang, Yayun Jiang, Jiayang Li, Minghui Wu, Guilian Wang, Yuanyuan Zhang, Meini Zhang

**Affiliations:** First Hospital of Shanxi Medical University, Taiyuan, China

**Keywords:** multiple sclerosis, serum protein, degree of disability, influencing factors, EDSS

## Abstract

**Objective:** To explore the factors influencing the degree of disability in patients with multiple sclerosis (MS), and to provide evidence for its early diagnosis, prognostic evaluation and clinical intervention.

**Methods:** This retrospective observational study included 72 patients with relapsing-remitting multiple sclerosis (RRMS) at the First Hospital of Shanxi Medical University. All patients completed craniocerebral and spinal cord MRI (with or without Gd enhancement) and were evaluated for Expanded Disability Status Score (EDSS) scores before receiving treatment.

**Results:** Among 72 patients with RRMS, 45 (62.5%) had an EDSS score ≤3; A total of 27 patients (37.5%) had an EDSS score >3 points. Univariate analysis showed that age, annual recurrence rate (ARR), drug use, albumin (ALB), triglycerides (TG), and total number of lesions in groups with EDSS score ≤3 were significantly different from those with an EDSS score > 3 points (*P* < 0.05). Multivariate logistic regression analysis showed that ALB, total number of lesions, and drug use were independent influencing factors of the degree of disability in patients with MS, and the difference was statistically significant (*P* < 0.05). An ROC curve was constructed using ALB and the total number of lesions. The AUC of ALB was 0.681, *P* < 0.05, and the best cut-off value was 44.2 g/L. Its sensitivity to predict the degree of disability in patients with multiple sclerosis was 85.2%, while its specificity was 51.1%. The AUC of the total number of lesions was 0.665 (*P* < 0.05) and the best cut-off value was 5.5. Its sensitivity to predict the degree of disability in patients with multiple sclerosis was 70.4%, while its specificity was 64.4%. The AUC of the combined ALB, total number of lesions, and drug use was 0.795 (*P* < 0.05), sensitivity was 77.8, and specificity was 73.3%. The optimal diagnostic cut-off value of the regression equation for the EDSS score of patients with multiple sclerosis was 0.420.

**Conclusion:** Serum ALB, total number of lesions, and drug use in patients with multiple sclerosis were independent factors influencing the degree of disability. These findings provide clinical evidence for the prognostic evaluation and early intervention of patients with multiple sclerosis.

## Introduction

Multiple sclerosis (MS) is a neurodegenerative autoimmune central nervous system disease characterized by inflammation, demyelination, and axonal degeneration (1). Most patients initially suffer from an intermittent disease, which evolves into a progressive disease over time. Focal inflammation is considered to be the basis of the onset and relapse-remitting period of MS, while axonal loss and neurodegeneration are the causes of progressive symptoms. Patients with MS may experience a decline in limb function in the early stage of the disease, which seriously affects the life and work ability, as well as significantly reduces their quality of life (2). Therefore, an in-depth study of factors influencing the degree of disability in patients with multiple sclerosis is of great significance for its early identification and prognosis assessment. At present, clinical studies on the factors affecting the degree of disability of MS patients are mostly focused on sex, age, course of disease, number of recurrences, and blood lipids, among others. There are few reports on the influence of serum protein levels on the degree of disability in MS patients. In recent years, many studies have reported that serum proteins may participate in the pathological mechanism of MS, and are related to the maintenance of inflammation and degree of disability (3). This study will further study the effect of serum protein levels on the degree of disability in MS patients on the basis of previous studies, and provide evidence for early clinical diagnosis and prognostic evaluation.

## Materials and Methods

### Subjects

The files of 72 RRMS patients admitted to the Department of Neurology, Shanxi Medical University First Hospital for MS between June 2015 and June 2021 were retrospectively reviewed. There were 27 men and 45 women, aged between 20 and 60 years old, with an average age of (41.79 ± 12.55) years old. The average EDSS score was 6.76 ± 3.23, with a minimum of 1 point and a maximum of 15 points. Inclusion criteria were patients diagnosed with RRMS according to the 2010 or 2017 McDonald diagnostic criteria, while the exclusion criteria were: (1) progressive course; (2) digestive system or metabolic disease; (3) malignant tumor and other wasting diseases, and (4) age <20 years old or more than 60 years old.

### Methods

#### Auxiliary Examination

All patients used 1.5T field strength instruments to perfect the head MRI (T1WI, T2WI, Gd-DTPA enhancement) and spinal cord MRI scans. A blood test for serum total protein (TP) (biuret endpoint method), albumin (ALB) (bromocresol green method), prealbumin (PAB) (turbidimetric method), triglycerides (TG) (enzymatic method: GPO-POD), total cholesterol (TC) (cholesterol oxidase method), high-density lipoprotein cholesterol (HDL) (direct method) and low-density lipoprotein cholesterol (LDL) (direct method) was also performed. All patients completed the EDSS assessment on the first day of admission and were divided into EDSS ≤ 3 and EDSS >3 groups.

#### Recording Data

General demographic data: gender, age, BMI, ARR and drug use; laboratory tests: four blood lipids (TC, TG, HDL, LDL), TP, ALB and PAB; Total number of cranial MRI lesions with low or equal signals on T1WI and high signals on T2WI (hereinafter referred to as the total number of lesions), number of Gd-enhanced lesions and number of spinal MRI lesions.

### Statistical Methods

SPSS22.0 statistical software was used for analysis. Quantitative data conforming to normal distribution were represented by mean ± standard $\bar{X}\pm S$ deviation, and comparison between groups was performed using a *t*-test; Quantitative data conforming to non-normal distribution was represented by the median and interquartile range. [M(P25-P75)] indicates that the Mann-Whitney U test was used for comparison between groups. The chi-square test was used to compare the qualitative data between the groups. Factors with statistically significant differences between the two groups were included in the multiple logistic regression analysis, and the model was established using stepwise regression. The inclusion criterion was 0.05, and the exclusion criterion was 0.10. The receiver operating curve (ROC) was used to determine the drug use, albumin, and the ability of the total number of lesions to diagnose the disease, while the Youden index was used to determine the best cut-off value (Yorden index = sensitivity + specificity −1). A *P* < 0.05 was used to indicate that the difference was statistically significant.

## Results

### Single Factor Analysis of Factors Affecting the Degree of Disability in Patients With MS

Among the 72 RRMS patients, 45 patients (62.5%) had an EDSS score ≤ 3, with an average age of (36.29 ± 11.27) years, including 19 men and 26 women, and with an average EDSS score of 5.16 ± 3.25; A total of 27 patients (37.5%) had an EDSS score > 3 points, with an average age of (42.59 ± 11.81) years, including 8 men and 19 women, with an average EDSS score of 6.78 ± 2.99 (Table 1). Univariate analysis showed that age, ARR, number of relapses, disease duration, drug use, ALB, TG, and total number of lesions in groups with an EDSS score ≤ 3 were significantly different from those with an EDSS score > 3 (*P* < 0.05).

**Table 1:** Univariate analysis of the degree of disability in MS patients.

**Index**	**≤3 (*n =* 45)**	**>3 (*n =* 27)**	***t*/*z*/χ^2^**	***P***
EDSS score	5.16 ± 3.25	6.78 ± 2.99	−2.11^a^	0.038^*^
Age	36.29 ± 11.27	42.59 ± 11.81	−2.257^a^	0.027^*^
Male/Female	19/26	8/19	1.142^b^	0.285
ARR	1.00 (1.00, 1.75)	0.73 (0.44, 1.00)	−2.554^c^	0.011^*^
Number of relapses	2.00 (1.00, 3.00)	4.00 (2.00, 8.00)	−3.602^c^	<0.001^**^
Disease duration	1.00 (1.00, 4.50)	8.00 (2.00, 13.00)	−3.815^c^	<0.001^**^
BMI	22.98 ± 4.05	22.39 ± 2.75	0.737^a^	0.464
Drug use [*n* (%)]	23 (51.1)	6 (22.2)	5.855^b^	0.016^*^
TP	67.61 ± 4.35	68.27 ± 5.11	−0.586^a^	0.560
Alb	44.05 ± 4.22	41.49 ± 4.22	2.498^a^	0.015^*^
PAB	278 ± 63.81	257.67 ± 75.39	1.222^a^	0.226
TC (mmol/L)	4.32 ± 0.98	4.25 ± 1.05	0.282^a^	0.779
TG (mmol/L)	0.92 (0.60, 1.58)	1.25 (1.02, 1.48)	−2.123^c^	0.034^*^
HDL (mmol/L)	1.16 ± 0.28	1.15 ± 0.33	0.130^a^	0.897
LDL (mmol/L)	2.53 ± 0.73	2.47 ± 0.92	0.299^a^	0.766
Total number of lesions	1.00 (1.00, 2.00)	2.00 (1.00, 2.00)	−2.841^c^	0.004^**^
Enhanced lesions [*n* (%)]	19 (42.2)	13 (48.1)	0.240^b^	0.624

* *P < 0.05*,

** *P < 0.01;*

a *: the t value of the independent sample t-test;*

b *: theχ^2^value of theχ^2^test;*

c *: the Z value of the Mann-Whitney U test. Univariate analysis showed that the age, ARR, number of relapses, disease duration, drug use, ALB, TG and total number of lesions in groups with an EDSS score ≤ 3 were significantly different from an EDSS score> 3 (P < 0.05)*.

### Multiple Logistic Regression Analysis of Factors Influencing the Degree of Disability in MS Patients

Patients with EDSS scores ≤ 3 points or EDSS scores > 3 were divided into dependent variables, and the statistically significant indicators in univariate analysis: age, ARR, number of relapses, disease duration, drug use, ALB, TG, and total number of lesions were independent variables for multivariate logistic regression analysis. On the other hand, ALB, total number of lesions, and drug use were independent influencing factors of the degree of disability in MS patients, with the difference between the factors being statistically significant (*P* < 0.05) (Table 2).

**Table 2:** Multiple logistic regression analysis of factors influencing the degree of disability in MS patients.

**Index**	**β**	**SE**	**Wald**	***P***	***OR***	***OR*** **95%**	
						**Lower** **limit**	**Upper** **limit**
Drug use	−1.529	0.622	6.051	0.014^*^	0.217	0.064	0.733
ALB	−0.171	0.069	6.138	0.013^*^	0.843	0.736	0.965
Total number of lesions	1.395	0.575	5.884	0.015^*^	4.035	1.307	12.457

* *P < 0.05; Multivariate logistic regression analysis found that ALB, total number of lesions and drug use were independent influencing factors of the degree of disability in MS patients, and the difference was statistically significant (P < 0.05)*.

### Construct ROC Curve Based on the Results of Logistic Model Analysis

**Figure 1:**
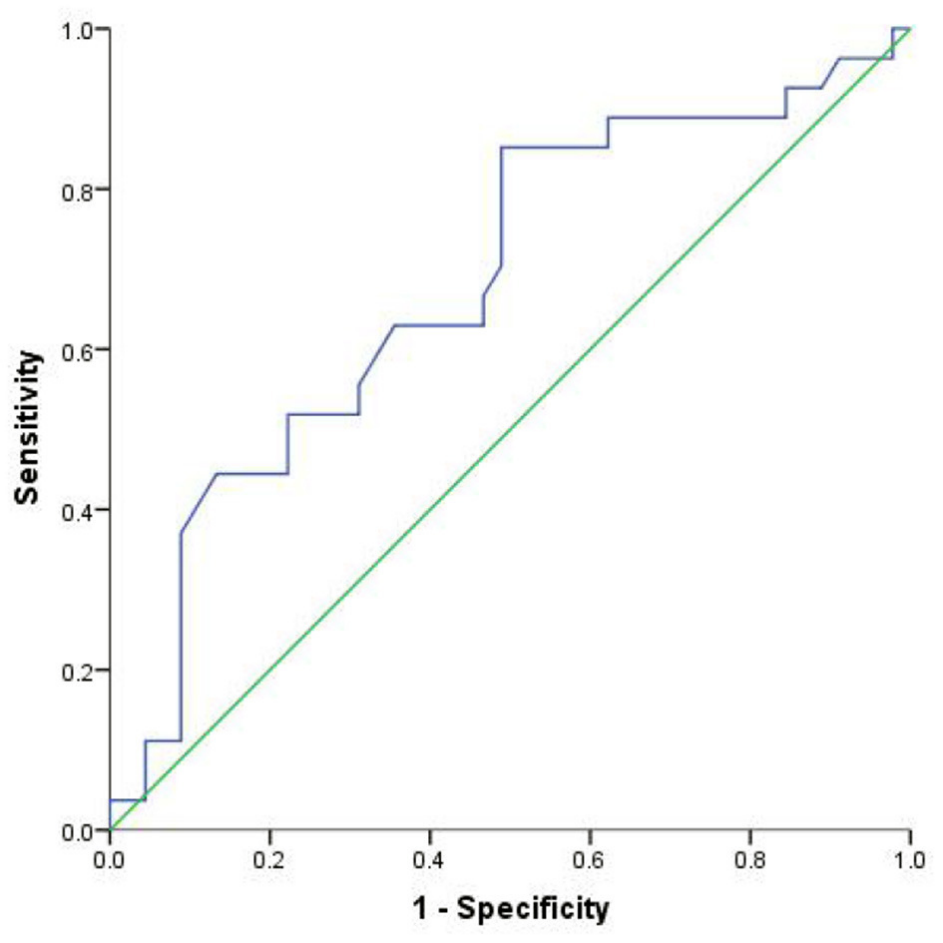
ROC curve of ALB.

**Figure 2:**
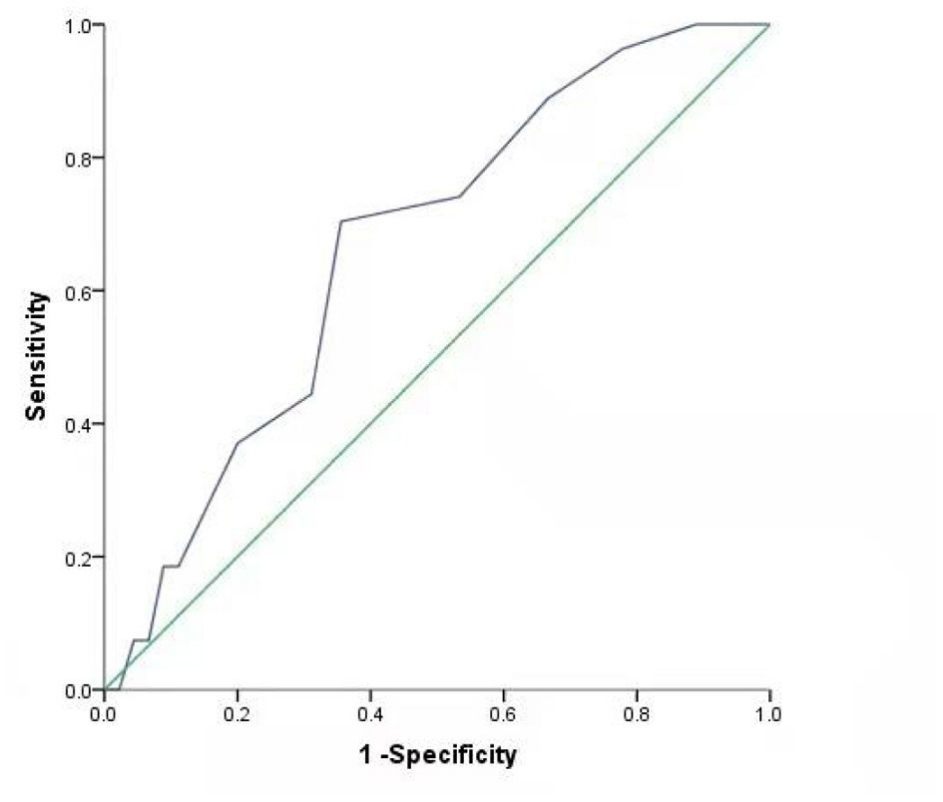
ROC curve of total number of lesions.

The ROC curve was constructed using ALB and the total number of lesions. The AUC of ALB was 0.681, *P* < 0.05, and the best cut-off value was 44.2 g/L. Its sensitivity in predicting the degree of disability in patients with multiple sclerosis was 85.2%, while its the specificity was 51.1%. The AUC of the total number of lesions was 0.665 (*P* < 0.05) and the best cut-off value was 5.5. Its sensitivity predicting the degree of disability in patients with multiple sclerosis was 70.4%, while its specificity was 64.4%. The AUC of the combined ALB, total number of lesions, and drug use was 0.795 (*P* < 0.05), sensitivity was 77.8, and specificity was 73.3%. The optimal diagnostic cut-off value of the regression equation for the EDSS score of patients with multiple sclerosis was 0.420 (Figures 1, 2, Table 3).


$$\begin{array}{l} \text{Combined diagnostic value}=\\ \frac{1}{1+e^{-6.606-1.719\times \text{Taked DMDs}-0.179\times ALB+0.189\times \text{Total number of lesions})}} \end{array}$$

**Table 3:** ROC analysis results of factors affecting the degree of disability in MS patients.

**Index**	**The best cut-off value**	**AUC** **(95% *CI*)**	**Sensitivity**	**Specificity**	***P***
ALB	44.200	0.681 (0.552, 0.811)	0.852	0.511	0.010^*^
Total number of lesions	5.500	0.665 (0.539, 0.791)	0.704	0.644	0.020^*^
Combined diagnosis	0.420	0.795 (0.693, 0898)	0.778	0.733	<0.001^**^

* *P < 0.05*,

** *P < 0.01; The AUC of ALB was 0.681, P < 0.05, and the best cut-off value was 44.2 g/L. Its sensitivity to predict the degree of disability in patients with multiple sclerosis was 85.2% and the specificity is 51.1%. The AUC of the total number of lesions was 0.665, P < 0.05, and the best cut-off value was 5.5. Its sensitivity to predict the degree of disability in patients with multiple sclerosis was 70.4%, and the specificity was 64.4%. The AUC of combined ALB, total number of lesions, and drug use was 0.795, P < 0.05, sensitivity was 77.8, and specificity was 73.3%. The optimal diagnostic cut-off value of the regression equation for the EDSS score of patients with multiple sclerosis was 0.420*.

## Discussion

Epidemiological studies report that the prevalence of MS in China is ~0.77–2.3%, and that male-to-female ratio is ~1:3.2 to 6.6 (4, 5). Physical disability is an important factor that affects the quality of life of patients with multiple sclerosis; therefore, it is particularly important to actively explore factors influencing the degree of physical disability. The results of this study show that the total number of lesions is an independent factor that influences the degree of disability in patients with multiple sclerosis. Harrison et al. using 7-T magnetic resonance imaging found that cortical lesion burden, including the number and volume of lesions, was closely related to physical disability and cognitive dysfunction in patients with multiple sclerosis (6). Later, another study using 3-T magnetic resonance imaging also came to the same conclusion (7). These results are consistent with the current study. A retrospective study in Egypt with a larger sample size was similar to this study, and divided the EDSS scores into three groups: 1–3, 3.5–6, However, the results were inconsistent with this study. The results of the study also showed that the number of TIWI and T2WI lesions was significantly correlated with the EDSS score (8). This may be related to the destruction of the integrity of the network connection structure in patients with MS (9). The AUC of the total number of lesions was 0.665 (*P* < 0.05), and the best cut-off value was 5.5. Its sensitivity predicting the degree of disability in patients with multiple sclerosis was 70.4%, while its the specificity was 64.4%. It is suggested that when the total number of lesions reaches 5.5, the risk of patients with EDSS ≥ 3 points is greater. This finding can provide a basis for clinical assessment of the disease degree and prognosis of patients.

Currently, more than a dozen disease-modifying therapies (DMTs) have been approved by the FDA for the treatment of relapsing-remitting multiple sclerosis. Studies have suggested that DMTs can reduce the risk of disability (10). This study found that patients with MS receiving DMTs had less limb disability. A long-term follow-up study by Healy et al. found that interferon-beta (IFN-β) could appropriately extend the time for EDSS to reach 4 points and 6 points (11). Batista et al. showed that fingolimod could significantly reduce the ARR of MS patients, and that the EDSS score remained stable during the use of fingolimod (12). Teliflunomide has a good effect on patients with mild disabilities (EDSS ≤ 2) initial treatment (13). Rituximab can reduce the ARR of RRMS and PPMS, as well as help maintain a stable or slightly improved EDSS score (14). These studies were consistent with the results of this study, suggesting that DMTs play an important role in reducing recurrence and delaying disease progression.

The results of this study show that serum ALB level is a significant factor influencing the degree of disability in patients with MS. Oliveira et al. found that ALB was a predictive factor for the diagnosis of multiple sclerosis, and that it was also a predictor for distinguishing RRMS from progressive multiple sclerosis (15). Studies have shown that ALB enters the central nervous system through damage of the BBB barrier damaged in patients with MS (16). Due to the high concentration of ALB in plasma, when the BBB leaks, ALB can easily enter the central nervous system from the blood circulation and produce beneficial and/or harmful effects. The beneficial effect is that ALB is a target of reactive oxygen species (ROS) and reactive nitrogen species (RNS), thereby limiting damage to other molecules. Moreover, ALB can also reduce the production of ROS and RNS by binding to iron and heme. Despite these protective properties, ALB may promote pathological progression by inducing the production of pro-inflammatory cytokines or disrupting potassium homeostasis, thereby making neurons more susceptible to the effects of glutamate excitotoxicity (17). ALB is a negative acute-phase reactant and constitutes an important extracellular antioxidant defense in the plasma. This protein has antioxidant properties, such as its ability to tightly bind copper and iron, and scavenge free radicals as well as, peroxynitrite. Oliveira et al. proposed that changes in the levels of TP and ALB in the acute phase may lead to redox imbalance in MS and lead to a burden of immune inflammation. The interaction between these factors causes a self-amplified feedforward loop, leading to a chronic state of immune inflammation and autoimmune pathways activation, which may promote the maintenance of MS. They also proposed that the reduction in ALB levels helps to distinguish RRMS from progressive MS (16). This may be the reason why the level of serum albumin affects the degree of disability in MS patients, but whether the decrease in serum ALB level is related to the change in cerebrospinal fluid ALB level requires further verification through experiments with a large sample size. The study also found that the AUC of albumin was 0.681 (*P* < 0.05), at the best cut-off value was 44.2 g/L. Its sensitivity to predict the degree of disability in MS patients was 85.2%, while its specificity was 51.1%. It is clear that when the ALB level is lower than 44.2 g/L, the risk of EDSS score > 3 points is greater. It suggests that clinicians should strengthen publicity and urge patients to improve their nutrition, increase high-quality protein intake to be able to delay disease progression.

The univariate analysis done in this study showed that serum TG level was an factor influencing the degree of disability in patients with MS, but not its independent influencing factor. Jorissen et al. also observed this correlation (18). Very low density lipoprotein (VLDL) rich in TG can promote the production of inflammatory cytokines (19), which drives more active monocytes to accumulate in the lesion site and further aggravate the demyelination (20). This may explain why TG affects the degree of disability in patients with MS. However, another study has also reported that TG were not related to the degree of disability in patients with MS (21). At present, there is still controversy about the relationship between TG and the degree of disability in patients with MS, so further exploration of large sample size, prospective clinical research, and basic research is needed.

The univariate analysis done in this study showed that the number of relapses, disease duration, and the ARR were all possible factors influencing the disability of MS patients. Incorporating ARR into a multivariate analysis, it was found that it was not an independent influencing factor of MS disability. Stewart et al. (22) conducted a prospective follow-up study on 136,462 patients with recurrent MS and found that a higher recurrence rate was related to greater disability accumulation, and a large number of basic studies are needed to further explore its possible mechanism.

The shortcomings of this study are as follows: (1) Since China is a region with a low incidence of multiple sclerosis, not many medical records were collected in this study within 5 to 6 years, which may have a certain impact on the results of the study; (2) This study was a retrospective study which failed to collect CSF-related protein indicators; (3) This study is cross-sectional in nature; thus, it was not possible to longitudinally detect changes in various indicators as the disease progressed. Therefore, the results of this study need to be further confirmed by prospective, longitudinal studies with a larger sample size.

In summary, the degree of disability in patients with multiple sclerosis is affected by many factors, and that there is not one single independent influencing factor influencing it. Close monitoring of related risk factors and early intervention may help improve the prognosis and quality of life of patients with multiple sclerosis.

## Data Availability Statement

The original contributions presented in the study are included in the article/Supplementary Material, further inquiries can be directed to the corresponding author/s.

## Author Contributions

MZ and YZ put forward research ideas and sets overall research goals. HX organizes and analyzes the data. ZY was responsible for writing the article. The remaining authors assisted in collecting the data and writing the article.

## Conflict of Interest

The authors declare that the research was conducted in the absence of any commercial or financial relationships that could be construed as a potential conflict of interest.

## Publisher's Note

All claims expressed in this article are solely those of the authors and do not necessarily represent those of their affiliated organizations, or those of the publisher, the editors and the reviewers. Any product that may be evaluated in this article, or claim that may be made by its manufacturer, is not guaranteed or endorsed by the publisher.
